# The Effectiveness of High-Intensity Focused Ultrasound in Treating Nasal Obstruction Caused by Inferior Turbinate Hypertrophy: A Clinical Study

**DOI:** 10.7759/cureus.58348

**Published:** 2024-04-15

**Authors:** Ali Muhssin Shnain Al-Hilali, Adnan Qahtan Khalaf, Ehab T Yaseen

**Affiliations:** 1 Department of Head and Neck Surgery - Otolaryngology, Southwest Jutland Hospital, Esbjerg, DNK; 2 Department of Head and Neck Surgery - Otolaryngology, Al-Yarmouk Teaching Hospital, Baghdad, IRQ; 3 Department of Head and Neck Surgery - Otolaryngology, Al-Yarmouk Teaching Hospital, College of Medicine, Mustansiriyah University, Baghdad, IRQ

**Keywords:** ultrasound volumetric tissue reduction (uvtr), nasal obstruction, inferior turbinate hypertrophy, high intensity focused ultrasound (hifu), cavitation phenomena, allergic rhinitis

## Abstract

Background

Nasal obstruction due to inferior turbinate hypertrophy is a common medical complaint among ENT clinic patients, which can significantly affect the patient's quality of life, and some are compelled to use topical intranasal decongestants. Conservative management is the first line of treatment; however, surgical reduction of the inferior turbinate becomes necessary if the symptoms persist after three months of treatment. The optimal surgical technique is controversial. High-intensity focused ultrasound (HIFU) is a minimally invasive surgical option that targets tissue volume precisely and minimally impacts surrounding tissue. This study aimed to assess the effectiveness and safety of HIFU in treating patients suffering from nasal obstruction due to inferior turbinate hypertrophy.

Methods

This prospective study was conducted from February to December 2016. The study lasted over six months. Patients with a history of allergic and non-allergic rhinitis participated in this study. It included 43 patients who had been experiencing chronic nasal obstruction due to bilateral inferior turbinate hypertrophy and had not shown improvement after three months of medical treatment. The patients underwent Ultrasound Volumetric Tissue Reduction (UVTR) surgery using the D & A Ultrasurg device (Diamant Medical Equipment Ltd., Amman, Jordan) under local anesthesia. The effectiveness, safety, and tolerance of HIFU were assessed subjectively for six months using a well-designed questionnaire utilizing a visual analog scale (VAS) and nasal endoscopy after the surgery.

Results

The study included 43 patients, 22 male and 21 female, aged 13 to 65 years. The study found that 40 (93%) patients showed significant improvement in nasal obstruction within a month of the surgery. However, three (7%) patients continued to experience persistent nasal obstruction even after six months of follow-up. The procedure was well-tolerated, with low rates of complications after surgery and reasonable pain control. During the surgery, 20 (46.5%) patients reported mild pain described as a pressure-like sensation, and 10 out of 43 patients (23%) required paracetamol after the procedure. Four patients (9.3%) had mild bleeding, which was treated with an ultrasound nasal probe without nasal packing. All patients experienced crusting of the nasal cavity during the first week, but no crustation was observed after the first month. There were no reported cases of synechia among the patients.

Conclusion

This study confirms that HIFU treatment is a reliable and effective treatment for improving short-term nasal obstruction caused by inferior turbinate hypertrophy. The procedure is easily applied and well-tolerated in outpatient clinics.

## Introduction

Nasal obstruction is a common complaint among patients who present to otolaryngologists, which disturbs the quality of life resulting from anatomical abnormalities or mucosal sinonasal diseases. Hypertrophy of the turbinate is one of the most common causes of nasal obstruction, and it is often seen in conditions such as allergic rhinitis and vasomotor rhinitis. Epidemiologic investigations have mentioned that up to 20% of the population of various countries has chronic nasal obstruction secondary to turbinate hypertrophy [1]. The first-line treatment for inferior turbinate hypertrophy is primarily conservative, and surgery is only indicated in refractory cases. Turbinate surgery aims to improve nasal breathing by reducing the size of hypertrophied turbinate while maintaining normal nasal physiology and avoiding complications. Despite the method used, most inferior turbinate procedures appeared to be effective in treating symptoms of nasal obstruction not relieved by medical therapy [1].

Inferior turbinate hypertrophy drastically impairs patients’ quality of life and obligates some to use topical intranasal decongestants [2]. No technique is perfect; each is associated with known short- and long-term complications, and the optimal method is controversial [3]. Surgery of the inferior turbinates has been reported as the eighth most common procedure performed by otolaryngologists [4].

There are various surgical techniques available for reducing the size of the inferior turbinates. These include total or partial inferior turbinectomy, lateral out fracture, turbinoplasty by microdebrider submucosal resection, laser-assisted turbinate reduction, cryosurgery, treatment with infrared light, vidian neurectomy, electrical coagulation, and ultrasound volume turbinate reduction (UVTR) [5]. A wider nasal cavity doesn't always mean better nasal function, and many authors now avoid total turbinectomy [6]. Ultrasound-based turbinate tissue volume reduction yields excellent results in our practice [7]. Ultrasound has traditionally only been used for laryngeal surgery [8], but more recently has been used to reduce tonsils [9].

This study aimed to evaluate the effectiveness and safety of high-intensity focused ultrasound (HIFU) in treating patients suffering from nasal obstruction caused by inferior turbinate hypertrophy. The study spanned a duration of six months.

## Materials and methods

Inclusion criteria

The study included male and female patients aged between 13 and 65. Patients with a history of allergic and non-allergic rhinitis participated in this study. They were suffering from bilateral mucosal inferior turbinate hypertrophy that did not respond to three months of conservative treatment with intranasal corticosteroid spray, antihistamine, and decongestant.

Exclusion criteria

Patients with chronic rhinosinusitis, nasal infection, septal deviation, and previous nasal surgery, including rhinoplasty, septal perforation, autoimmune disease, benign and malignant sinonasal tumors, and facial anomalies, were excluded.

Patients, study design, and location

This prospective study was conducted from February 2016 to December 2016 at an outpatient clinic in Baghdad, Iraq. All patients provided informed consent after being informed about the procedure and its potential benefits and risks. The study enrolled 43 patients (21 females and 22 males) who were followed up for 6 months. The clinical assessment before the surgery involved a thorough history, an ENT physical examination, and a rigid sinuscope. The hypertrophy of the mucosa in the inferior turbinate was confirmed by the noticeable reduction observed after the application of xylometazoline nasal drops. The nasal endoscopy assessment was performed by Karl Storz rigid sinuscope (KARL STORZ, Tuttlingen, Germany) 0°, 4 mm, 18 cm under local anesthesia. The patients were instructed to complete a questionnaire using the visual analog scale (VAS) before and after the surgery. VAS is a scale from 0 to 10 that measures nasal obstruction, where 0 is no obstruction and 10 is complete obstruction [10]. The patients completed a VAS to record the level of nasal obstruction before the surgery (Table 1). We conducted clinical and nasal endoscopic examinations at intervals of one week, one month, three months, and six months. We graded the inferior turbinate using the Friedman grading system (Table 2).

**Table 1:** Evaluation questionnaire form using a visual analog scale

Symptoms	No problem	Mild	Moderate	Severe
Nose obstruction	0	1-3	4-6	7-10
Pain	0	1-3	4-6	7-10
Bleeding	0	1-3	4-6	7-10
Crustation	0	1-3	4-6	7-10

**Table 2:** The endoscopic grading system of the inferior turbinate

Grade	
Grade Ⅰ	The turbinate was defined as mild enlargement with no obvious obstruction
Grade Ⅱ	The turbinate was in between grade I and grade III
Grade Ⅲ	The turbinate completely occluded the nasal cavity

Endoscopic grading of inferior turbinate

There is no universally standardized system for scoring turbinate hypertrophy [11]. The Friedman grading system graded inferior turbinate by anterior rhinoscopy and nasal endoscopy [12].

The Ministry of Food and Drug Safety has granted approval for the use of the D & A Ultrasurge device (Diamant Medical Equipment Ltd., Amman, Jordan) in the study conducted. It is compliant with all current European legislation for manufacturing and trading medical equipment. It is also certified with ISO 9001:2008 and ISO 13485:2012. The study was approved by the Institutional Review Board of the Iraqi Board for Medical Specialties in Baghdad, Iraq (Ref: 4502).

The procedure

**Figure 1:**
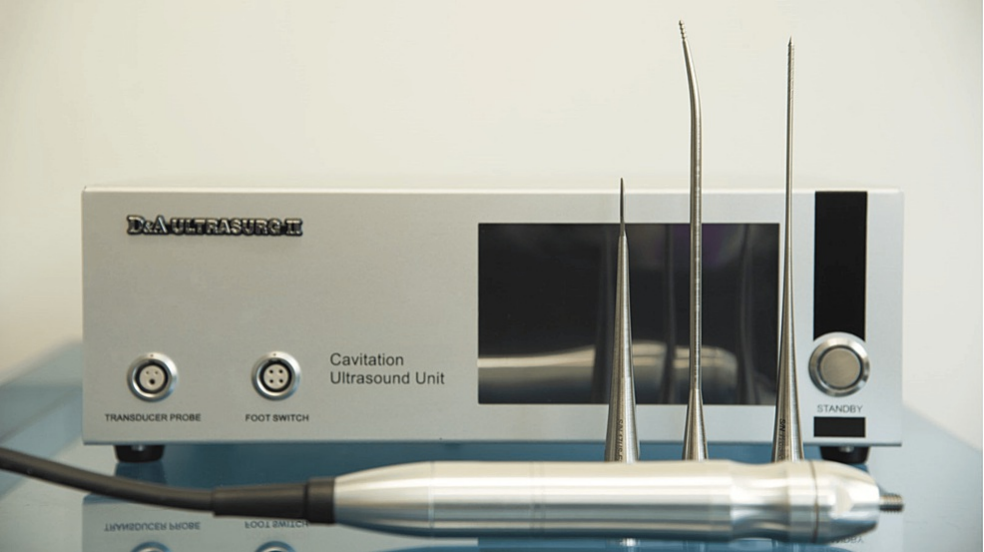
D & A Ultrasurg ultrasound device including the central unit, footswitch, transducer, and probes

**Figure 2:**
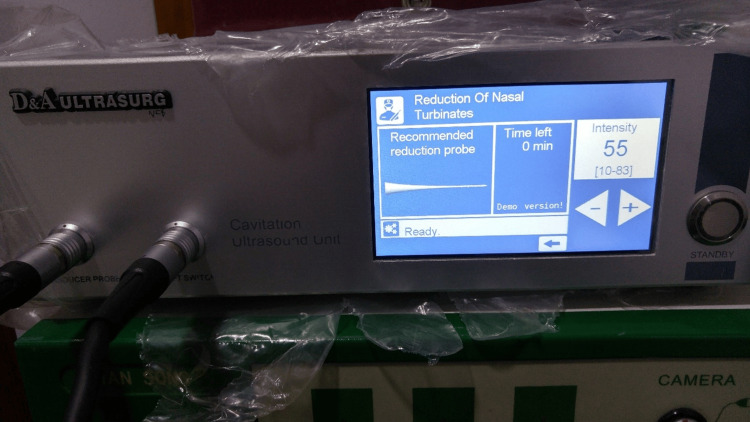
D & A Ultrasurg ultrasound device allows the adjustment of intensity via a touch monitor The intensity used in the conducted study was 55.

**Figure 3:**
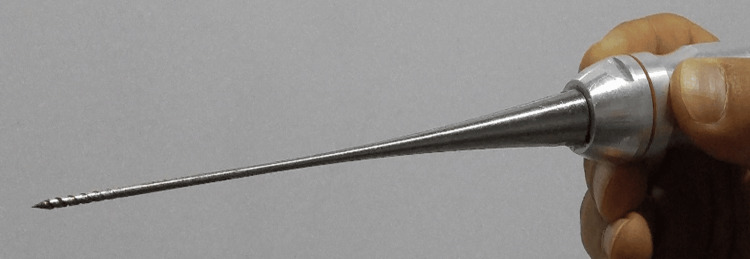
A reusable transducer nasal probe with a pointed end made of specialized aluminum alloy eliminates temperature issues for operators

The surgery was performed using a D & A Ultrasurg device (Diamant Medical Equipment Ltd.) (Figure 1). We performed all the procedures in an outpatient clinic with local anesthesia, using cotton strips soaked with 10% lidocaine and xylometazoline for 10 minutes. No medication was given before the procedure. The pain during surgery was assessed using the visual analog scale and categorized as none, mild, moderate, and severe. The procedure was performed while the patient lay supine with a 15° head up. We adjusted the intensity of the ultrasound generator to 55 (Figure 2). With a rigid sinuscope guide, we examined the external surface of the inferior turbinate for any pain sensation. After the inspection, we introduced the nasal probe into the turbinate head submucosally. The nasal probe was advanced slowly using light pressure toward the posterior end of the inferior turbinate. Then, the device was activated for about 10-20 seconds until the desired shrinkage was achieved with the assistance of a rigid sinuscope. Following this, the probe was slowly removed (while the device was activated continuously), while pausing briefly at intervals of 10 mm length every 2-3 sec while checking the nasal concha simultaneously. Hemostasis was achieved on inertance sites using an ultrasound nasal probe (Figure 3). We created two to three tunnels in various locations of the inferior turbinate. The number of tunnels and their specific sites depended on the desired level of shrinkage (Video 1). The ultrasound probe was cleaned and sterilized by immersion in an activated glutaraldehyde solution (CIDEX) containing 2.45% glutaraldehyde while the probe was activated.

**Video 1:** Endoscopic reduction of the inferior turbinate using the ultrasound volumetric tissue reduction (UVTR) technique and follow-up intervals

The postoperative assessment included observation for about one hour for any bleeding or other complications. No patients needed nasal packing or antibiotic therapy after the surgery, and no major postoperative complications were reported. The patients were discharged home after one hour of observation. They were advised to rinse their nose three times daily for four weeks and to take paracetamol if needed.

During the one-week visit, all patients had crustation, which was cleared by suction (Video 1). The patients completed follow-up assessments, including the questionnaire using a VAS score, and clinical and nasal endoscopic examinations at one, three, and six months, taking into account the potential swelling and edema during the first three weeks after surgery.

Statistical analysis

We used the SPSS 22 (Statistical Packages for Social Sciences version 22) statistical package for statistical analysis (IBM Corp., Armonk, NY, US). The data were presented using basic measures of frequency and percentage [13].

## Results

In this study, 43 patients aged between 13 and 65 years participated, out of which 22 were males (51.1%) aged between 19 and 65 years, and 21 were females (48.8%) aged between 13 and 58 years (Table 3). During surgery, 20 patients (46.5%) reported mild pain, which they described as a pressure-like sensation. Only 10 out of 43 patients (23%) required paracetamol after the surgery (Table 4). The procedure was well-tolerated, with mean VAS scores obtained during the first hour after surgery. Only a minority of the patients, i.e., 4 out of 43 (9.3%), exhibited mild postoperative bleeding. However, the ultrasound nasal probe achieved hemostasis without nasal packing (Table 4). All patients noticed crusting of the nasal cavity during the one week following the procedure; however, no crustation was seen after one month (Table 4).

**Table 3:** Patient age groups distributed by gender Data presented as numbers and percentages

Age group (years)	Male	%	Female	%
< 20	1	12.5	7	87.5
20-29	4	57.1	3	42.9
30-39	8	66.7	4	33.3
40-49	4	57.1	3	42.9
50-59	2	33.3	4	66.7
= >60	3	100		
Total	22	51.2	21	48.8

**Table 4:** Complications during and after surgery Data presented as numbers and percentages using the visual analog scale (VAS)

Variable	Intraoperative	1^st^ week	1^st^ month	3^rd^ month	6^th^ month
Pain	20 (46.5%)				
Bleeding	4 (9%)				
Crustation		43 (100%)			

During the one month of follow-up, 40 out of 43 patients (93%) experienced a significant improvement in nasal obstruction, with only three patients (7%) still experiencing persistent nasal obstruction (Table 5). The size of the inferior turbinate was significantly reduced in 40 patients (93%), as confirmed by nasal endoscopy. However, three patients (7%) showed grade II hypertrophy. During the three-month follow-up period, three patients (7%) still experienced persistent nasal obstruction, with two patients (4.65%) showing grade II hypertrophy and one patient (2.22%) showing grade III hypertrophy on nasal endoscopy (Table 6). During the six-month follow-up period, out of 43 patients, 40 showed improvement in nasal obstruction while the remaining 3 (7%) still experienced persistent nasal obstruction (Table 5).

**Table 5:** Nasal obstruction before and after surgery Data presented as numbers and percentages using the visual analog scale (VAS)

Period	No obstruction	Mild	Moderate	Severe
Preoperative		2 (4.7%)	11 (25.6)	30 (56.6%)
1^st^ week	1 (2.3%)	2 (4.7%)	9 (21%)	31 (72 %)
1^st^ month	40 (93%)	3 (7%)		
3^rd^ month	40 (93%)		3 (7%)	
6^th^ month	40 (93%)		2 (4.7%)	1 (2.3%)

**Table 6:** Endoscopic grades of inferior turbinate before and after surgery Data are presented as numbers and percentage

Nasal endoscopy	Preoperative	1^st^ week	1^st^ month	3^rd^ month	6^th^ month
Inferior turbinate grade Ⅰ			40 (93%)	40 (93%)	40 (93%)
Inferior turbinate grade Ⅱ	20 (46.5%)		3 (7%)	2 (4.7%)	2 (4.7%)
Inferior turbinate grade Ⅲ	23 (53.5%)			1 (2.3%)	1 (2.3%)

## Discussion

Different types of turbinate surgeries are commonly used for treating nasal obstruction, but the best method is still a matter of debate. Each method's efficacy should be evaluated subjectively and objectively by assessing the degree of reduction in nasal obstruction, as well as the short-term and long-term side effects. Submucosal techniques have advantages in preserving overall nasal physiology because they destroy some of the sizeable cavernous blood spaces or sinusoids of the inferior nasal turbinate, which leads to the shrinkage of its mucous membrane. This initiates the formation of fibrous tissue that attaches the mucosa to the periosteum [14].

Chronic nasal obstruction caused by inferior turbinate hypertrophy can be challenging to manage, regardless of the underlying cause. Although various approaches exist, no "gold standard" method exists [15].

The HIFU technique utilizes mechanical power to reduce submucosal tissue. This is achieved by creating cavities caused by exposing the affected tissues to ultrasonic disintegration through a submucosal low-frequency fluctuation delivered by an ultrasonic nasal probe, which explodes the cells when combined with low pressure at the end of the waveguide. This leads to tissue separation and detachment at various levels, a phenomenon known as cavitation. Ultrasound can induce mechanical, thermal, and cavitation effects in the targeted tissue. Increasing local temperatures to 70 °C-100 °C can cause protein coagulation and tissue necrosis [16].

Ultrasound has been used in general surgery since the 1960s, but it has only recently gained attention in the ENT specialty, particularly in nasal surgery. Fast restoration of nasal and respiratory function can be achieved through this method. The use of ultrasound in rhinology is new and requires adequate documentation in the literature [17].

Our study prospectively evaluates the effectiveness of HIFU in treating nasal obstruction caused by hypertrophied nasal turbinates.

In our study, we found that males were more likely to have inferior turbinate hypertrophy(Table 3), which is consistent with previous research by Gindros et al. (2010). Their study included 60 patients, with a mean age of 32.2 ± 11.1 years (range 35-65 years); 28 were female (46.7%) and 32 were male (53.3%) [17].

All 43 patients in our study experienced some form of nasal obstruction, with most of them being severely affected before the procedure. On the one-week visit after surgery, most patients experienced worsened or persistent nasal obstruction; this might be due to the accumulation of crusts, congestion, and swelling following surgery. Many patients reported severe symptoms, some had mild to moderate symptoms, and one patient (2.3%) had no obstruction (Table 5). Crusting of the nasal cavity was found in all 43 patients at the site of an ultrasound nasal probe insertion. However, no crustation was observed during the one-month, three-month, and six-month visits (Table 4).

On the first month visit, our research showed that there was a significant improvement in nasal obstruction, and this improvement lasted for six months. Out of 43 patients, only three (7%) reported nasal obstruction during the three-month visit. By the 6-month visit, 40 patients (93%) reported no nasal obstruction, while only 3 patients (7%) continued to experience persistent nasal obstruction (Table 5). These findings are consistent with the study conducted by Gindros et al. (2010), who also observed a significant improvement in nasal obstruction during the first month after surgery [17].

We evaluated the inferior turbinate before surgery using an endoscopic grading system. Our study found that 20 out of 43 patients (46.5%) had grade II, while the remaining 23 patients (53.5%) had grade III (Table 6). There was a significant improvement in the size of the inferior turbinate during different periods after surgery. During the 6-month visit, out of 43 patients, 40 patients (93%) had grade Ⅰ, only two patients (4.7%) had grade II, and one patient (2.3%) had grade III. This outcome shows a strong association with nasal obstruction using the VAS score outlined in the study (Table 5).

Our study found that all patients underwent the procedure smoothly without any significant complications during or after the surgery (Table 4). These results are comparable to the study of Gindros et al. (2010), who found a low incidence of minor complications and no moderate or significant complications [17].

Our study demonstrated that 20 patients (46.5%) experienced mild pain during the procedure. However, no pain was reported in any patients during the one-week visit after surgery (Table 4). Gindros et al. (2010) noted that postoperative pain was mild and treated with an acetaminophen tablet [17].

According to our study's results, only four patients (9.3%) experienced mild bleeding during the surgery. The bleeding was quickly and effectively treated using an ultrasound nasal probe to achieve hemostasis at the bleeding site; no patient required nasal packing (Table 4). Gindros et al. (2010) mentioned that one patient experienced minor bleeding on the fourth day after surgery and was efficiently treated with bipolar electrocautery [17]. All patients had no synechia; these results were compatible with Gindros et al. (2010) [17].

Patients who did not show improvement after six months of follow-up post-surgery were recommended to start using intranasal corticosteroids.

Limitations of the study

Evaluating the nasal obstruction before and after surgery was challenging without objective measures such as rhinometry. Additionally, the follow-up period was limited to just six months, which needed to be increased to assess the long-term effects of HIFU.

## Conclusions

This clinical result found that high-intensity focused ultrasound treatment of a nasal obstruction caused by hypertrophic inferior nasal turbinates due to chronic allergic and non-allergic rhinitis can be considered safe, with minor discomfort and minimal risk of side effects. The procedure is fast, has negligible discomfort, and doesn't require nasal packing postoperative, with satisfactory results. Additionally, it is a highly effective treatment option that produces outstanding outcomes, lasting at least six months. We recommend further studies based on objective measures like rhinomanometry and acoustic rhinometry. Furthermore, conducting additional research can help validate the long-term impact of high-intensity focused ultrasound.
